# A Low Molecular Weight Hyaluronic Acid Derivative Accelerates Excisional Wound Healing by Modulating Pro-Inflammation, Promoting Epithelialization and Neovascularization, and Remodeling Collagen

**DOI:** 10.3390/ijms20153722

**Published:** 2019-07-30

**Authors:** Yin Gao, Yao Sun, Hao Yang, Pengyu Qiu, Zhongcheng Cong, Yifang Zou, Liu Song, Jianfeng Guo, Tassos P. Anastassiades

**Affiliations:** 1 Key Laboratory for Molecular Enzymology and Engineering of Ministry of Education, School of Life Sciences, Jilin University, Changchun 130012, China; 2 School of Pharmaceutical Sciences, Jilin University, Changchun 130021, China; 3 Departments of Medicine (Div. of Rheumatology), and of Biomedical and Molecular Sciences, Queen’s University, Kingston, ON K7L 3N6, Canada

**Keywords:** hyaluronan, *N*-butyrylation, anti-inflammation, angiogenesis, lymphangiogenesis

## Abstract

Recent knowledge of the cellular and molecular mechanisms underlying cutaneous wound healing has advanced the development of medical products. However, patients still suffer from the failure of current treatments, due to the complexity of healing process and thus novel therapeutic approaches are urgently needed. Previously, our laboratories produced a range of low molecular weight hyaluronic acid (LMW-HA) fragments, where a proportion of the glucosamine moieties were chemically N-acyl substituted. Specifically, *N*-butyrylation results in anti-inflammatory properties in a macrophage system, and we demonstrate the importance of N-acyl substituents in modulating the inflammatory response of LMW-HA. We have set up an inter-institutional collaborative program to examine the biomedical applications of the *N*-butyrylated LMW-HA (BHA). In this study, the potentials of BHA for dermal healing are assessed in vitro and in vivo. Consequently, BHA significantly promotes dermal healing relative to a commercial wound care product. By contrast, the “parent” partially de-acetylated LMW-HA (DHA) and the re-acetylated DHA (AHA) significantly delays wound closure, demonstrating the specificity of this N-acylation of LMW-HA in wound healing. Mechanistic studies reveal that the BHA-mediated therapeutic effect is achieved by targeting three phases of wound healing (i.e., inflammation, proliferation and maturation), demonstrating the significant potential of BHA for clinical translation in cutaneous wound healing.

## 1. Introduction

Wound healing generally proceeds efficiently after the onset of a lesion, however, a poor outcome may follow larger injuries or a variety of pathological states, such as infection and vascular disease [1,2]. Impaired cutaneous wound healing may become life-threatening, and is a major public health issue worldwide [3]. Recently, novel regenerative and reparative therapies have been developed [4], including the administration of growth factors [5], cell reprogramming [6] and tissue engineering [7].

However, several hurdles remain for the application of aforementioned approaches to cutaneous lesions; for example, the administration of growth factors lacks appropriate drug delivery systems [8], and the efficacy of cell-based strategies may be dampened by complexities, including the pathological conditions of donors, the onset time and duration of treatment, and the dose and its route of administration [9]. Therefore, viable and efficient alternatives are still needed.

It is known that the biological activities of hyaluronic acid (HA, a glycosaminoglycan of the extracellular matrix (ECM)) differ, depending on the molecular weight [10,11]. For example, high molecular weight HA (HMW-HA) possesses anti-inflammatory or immunosuppressive activities, while low molecular weight HA (LMW-HA) demonstrates pro-inflammatory or immunostimulatory behaviors [12]. In addition, HMW-HA displays anti-angiogenic properties, whereas LMW-HA is able to promote the formation of new blood vessels [13]. Although the potential of HA in wound healing is demonstrated preclinically in animals [14,15,16,17], there have been no reports that HA may fully promote different phases of wound repair.

In a previous communication [18] we had produced a range of LMW-HA polymers (30 to 214 kDa) by partial N-deacetylation with hydrazinolysis or NaOH treatment of the “parent”, intact HMW-HA. The resultant LMW-HA polymers with ~20% free NH_2_ functional groups were further acylated using acyl anhydrides. The partially *N*-butyrylated derivative, BHA, demonstrates a suppression of the stimulation of pro-inflammatory cytokine production by cultured human macrophages. Also, we showed the critical role of the N-acyl groups in the glucosamine moieties of LMW-HA for modulating the pro-inflammatory response, likely through the TLR-4 receptor system [18]. In the current study, the healing efficacy of BHA is assessed in rats with excisional full-thickness wounds, and the healing mechanisms are investigated.

## 2. Results

### 2.1. N-butyrylated LMW-HA (BHA) Improves Murine Cutaneous Wound Healing

BHA (~39 kDa, containing 0% NH_2_, 82.2 ± 4.6% *N*-acetyl, and 22.7 ± 3.8% *N*-butyryl moieties), DHA (~42 kDa, containing 21.6 ± 1.1% NH_2_, 78.4 ± 0.6% *N*-acetyl, and 0.0% *N*-butyryl moieties) and AHA (~45 kDa, containing 0.0% NH_2_, 97.7 ± 1.4% *N*-acetyl, and 0.0% *N*-butyryl moieties) (Figures S1 and S2) were assessed for their healing rates in rats with excisional full-thickness wounds. The gel formulation (BHA-Gel, DHA-Gel and AHA-Gel) (see Supporting Information) was applied to release LMW-HA derivatives locally into wounds. Results demonstrated that the wound closure efficacy was significantly improved by BHA-Gel at doses from 0.05 to 1 mg/mL relative to the untreated group (*p* < 0.05 and *p* < 0.01) (Figure S3). In contrast, the DHA-Gel and AHA-Gel ranging at the same doses significantly delayed the dermal wound closure relative to the untreated group (*p* < 0.05, data not shown). Due to the effective wound closure achieved by the BHA-Gel at the dose of 0.25 mg/mL (*p* < 0.01) at all tested timepoints (Figure S3), this concentration was chosen for the following in vivo experiments.

**Figure 1:**
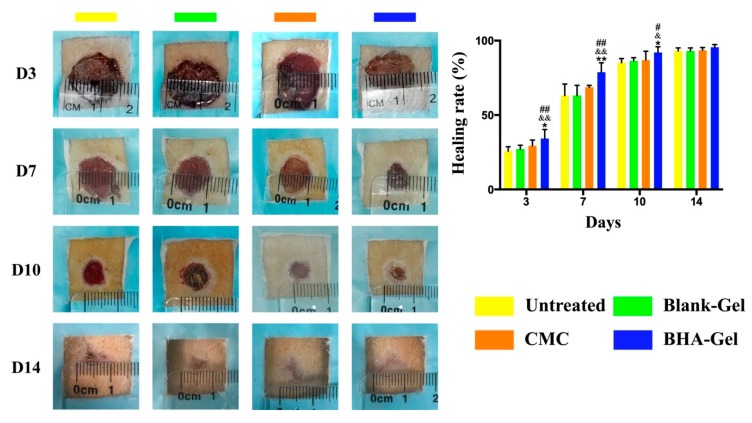
Representative images of incisional wounds in rats after treatments. Healing rate (%) when compared with the wound area on Day 0 (*n* = 6). # *p* < 0.05 and ## *p* < 0.01 relative to the untreated control group; & *p <* 0.05 and && *p <* 0.01 relative to Blank-Gel; * *p* < 0.05 and ** *p* < 0.01 relative to carboxymethyl chitosan (CMC).

**Figure 2:**
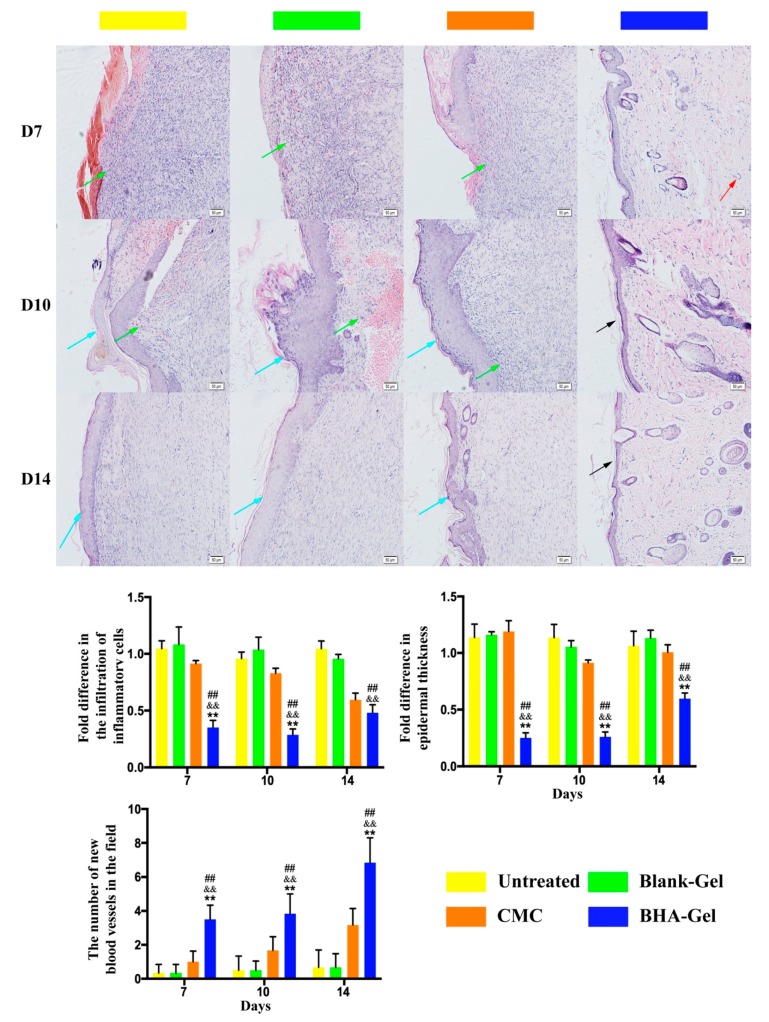
In representative hematoxylin-eosin (H&E) staining images (×100, bar in the lower right corner = 50 μm), green, blue, red and black arrows represent pro-inflammatory cells, epidermal hyperplasia, new blood vessel and intact epidermal structure, respectively. The pro-inflammatory cells, epidermal hyperplasia, and new blood vessel were quantified (*n* = 6). ## *p* < 0.01 relative to the untreated control group; && *p <* 0.01 relative to Blank-Gel; ** *p* < 0.01 relative to CMC.

As shown in Figure 1, the BHA-Gel significantly promotes the wound closure in comparison to the untreated control group and the Blank-Gel (*p* < 0.05 and *p* < 0.01). The closure rate is also significantly improved by BHA-Gel relative to the carboxymethyl chitosan (CMC, the active ingredient of a commercial wound care product, CHITIN®) (*p* < 0.05 and *p* < 0.01). In contrast, DHA-Gel and AHA-Gel significantly delay the wound closure relative to these control groups (*p* < 0.05 and *p* < 0.01) (Figures S4 and S5). In addition, reduced infiltration of pro-inflammatory cells (green arrows) and epidermal hyperplasia (blue arrows) were evident in BHA-Gel group from Day 7 to Day 14, when compared to other controls (Figure 2). The skin layer is more organized following the BHA-Gel treatment, which is accompanied with the development of discrete skin structures, including blood vessels (red arrows) and epidermal integrity (black arrows) (Figure 2). No significant difference was observed between the untreated control group and Blank-Gel (Figure 2), confirming that the wound closure results from BHA (Figure 1).

### 2.2. BHA Suppresses the MAPK and NF-κB Signaling Pathways

**Figure 3:**
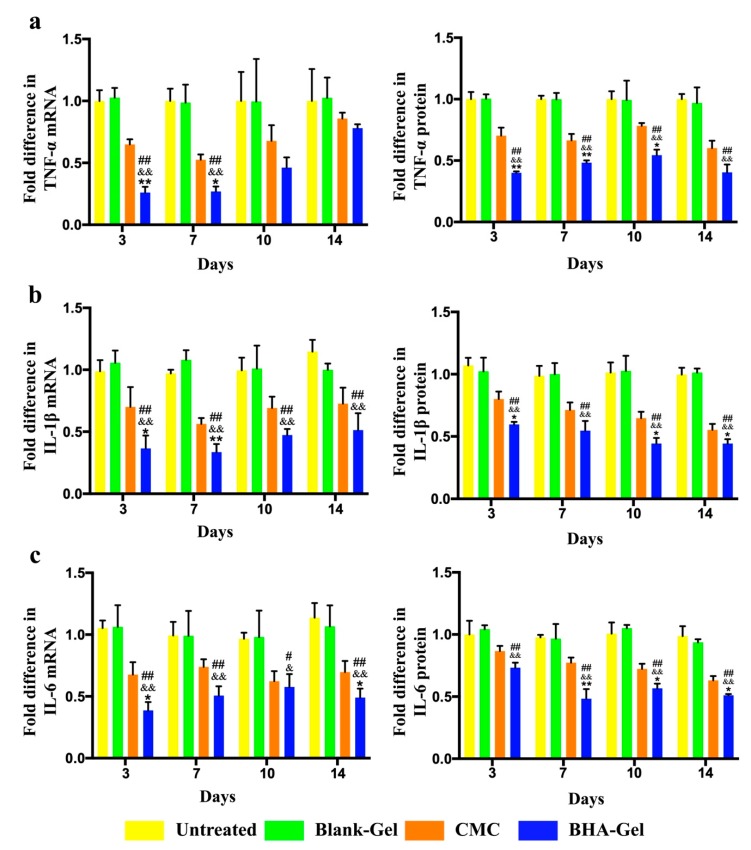
The mRNA and protein levels of (**a**) tumor necrosis factor-α (TNF-α), (**b**) IL-6 and (**c**) interleukin-1β (IL-1β) in wounds are determined by reverse transcription polymerase chain reaction (RT-PCR) and enzyme-linked immunosorbent assay (ELISA), (*n* = 6). # *p* < 0.05 and ## *p* < 0.01 relative to the untreated control group; & *p <* 0.05 and && *p <* 0.01 relative to Blank-Gel; * *p* < 0.05 and ** *p* < 0.01 relative to CMC.

As shown in Figure 3a, the tumor necrosis factor-α (TNF-α) mRNA and protein levels in wounds are determined using the reverse transcription polymerase chain reaction (RT-PCR) and the enzyme-linked immunosorbent assay (ELISA), indicating that BHA-Gel significantly reduces the TNF-α production (*p* < 0.01). Similarly, the expression of interleukin-1β (IL-1β) and IL-6 is also significantly reduced by this BHA-Gel in injured samples (*p* < 0.01) (Figure 3b,c). In contrast, the cytokine production is significantly elevated by the DHA-Gel (Figure S6) (*p* < 0.01) and AHA-Gel (Figure S7) (*p* < 0.01).

**Figure 4:**
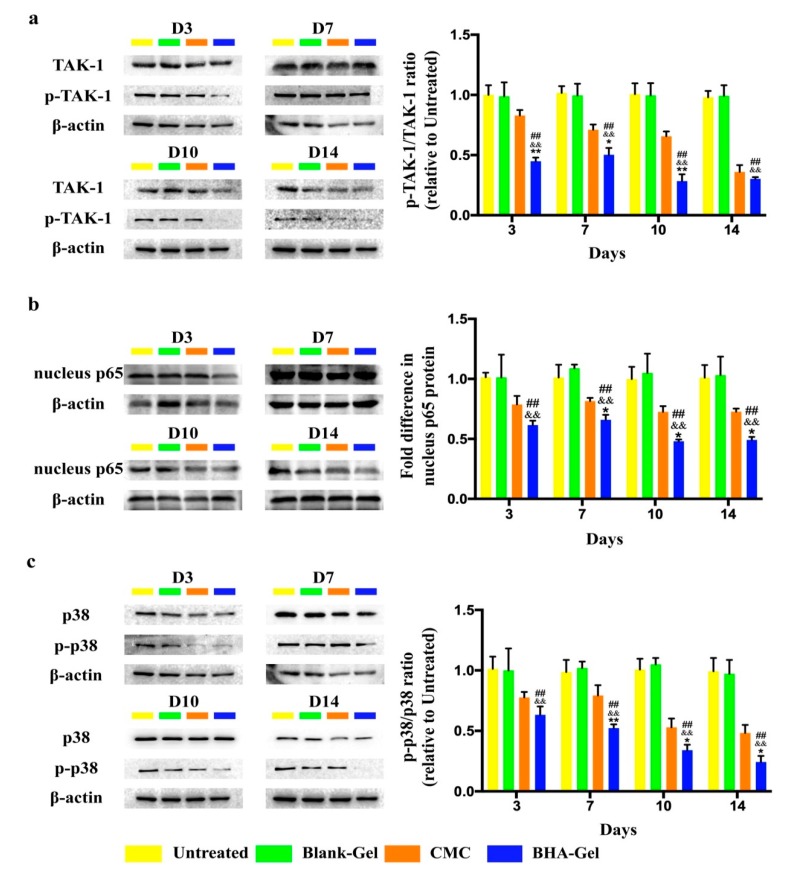
The protein level of (**a**) TGF-β activated kinase 1 (TAK-1) and phosphorylated TAK-1, (**b**) nuclear-p65, (**c**) p38 and phosphorylated p38 is determined using western blotting and quantified (*n* = 6). ## *p* < 0.01 relative to the untreated control group; && *p <* 0.01 relative to Blank-Gel; * *p* < 0.05 and ** *p* < 0.01 relative to CMC.

The expression of Mitogen-activated protein kinase, kinase 7 (MAP3K7, also known as TGF-β activated kinase 1, TAK-1) protein (including the phosphorylated form) in wounds is evaluated using western blotting (Figure 4a), indicating that BHA-Gel significantly inhibits the expression of phosphorylated TAK-1 protein (*p* < 0.01). As a result, the expression of nuclear-p65 protein (a gene product from the nuclear factor-κB (NF-κB) transcription factor complex; the transportation of p65 protein into nucleus activates the NF-κB pathway) is significantly reduced accordingly (*p* < 0.01) (Figure 4b). In addition, the activation of p38 MAP kinase, an important member of the mitogen-activated protein kinases (MAPK) family, is significantly suppressed by BHA-Gel (*p* < 0.01) (Figure 4c). These results suggest that BHA, but not DHA or AHA, can reduce the pro-inflammatory cytokine production *in vivo*, and BHA-mediated anti-inflammatory functions are partially due to the modulation of NF-κB and MAPK signal cascades, which may likely promote cutaneous wound healing during the inflammation phase.

### 2.3. BHA Promotes Re-Epithelialization, Angiogenesis and Lymphangiogenesis

**Figure 5:**
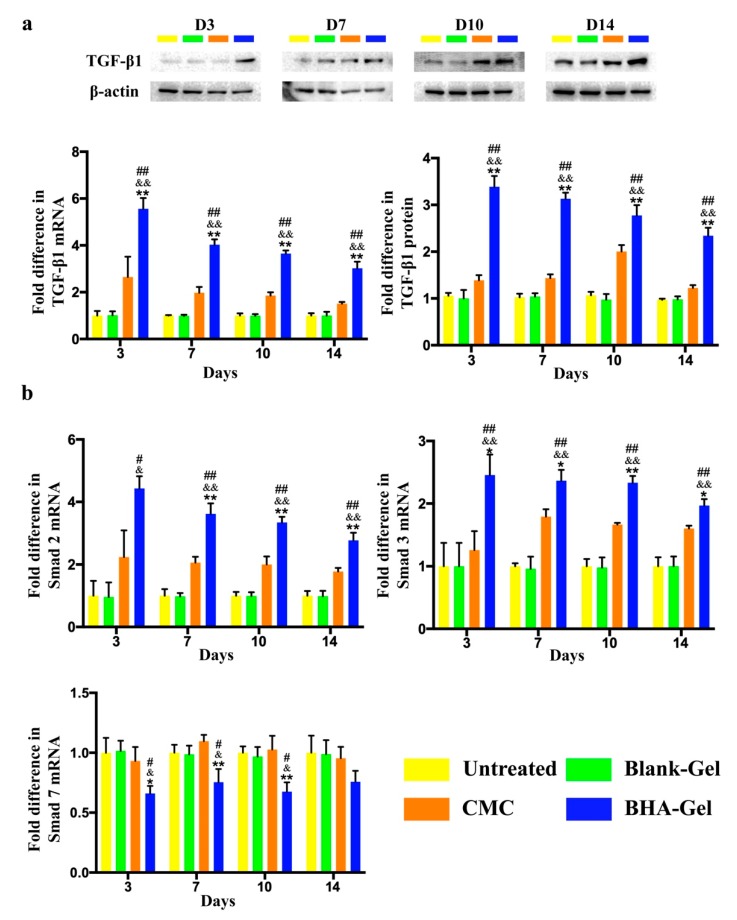
(**a**) The transforming growth factor beta 1 (TGF-β1) mRNA and protein levels in wounds are determined by RT-PCR and western blotting, respectively (*n* = 6). (**b**) The mRNA level of Smad2, Smad3 and Smad7 is determined by RT-PCR (*n* = 6). # *p* < 0.05 and ## *p* < 0.01 relative to untreated control group; & *p <* 0.05 and && *p <* 0.01 relative to Blank-Gel; * *p* < 0.05 and ** *p* < 0.01 relative to CMC.

**Figure 6:**
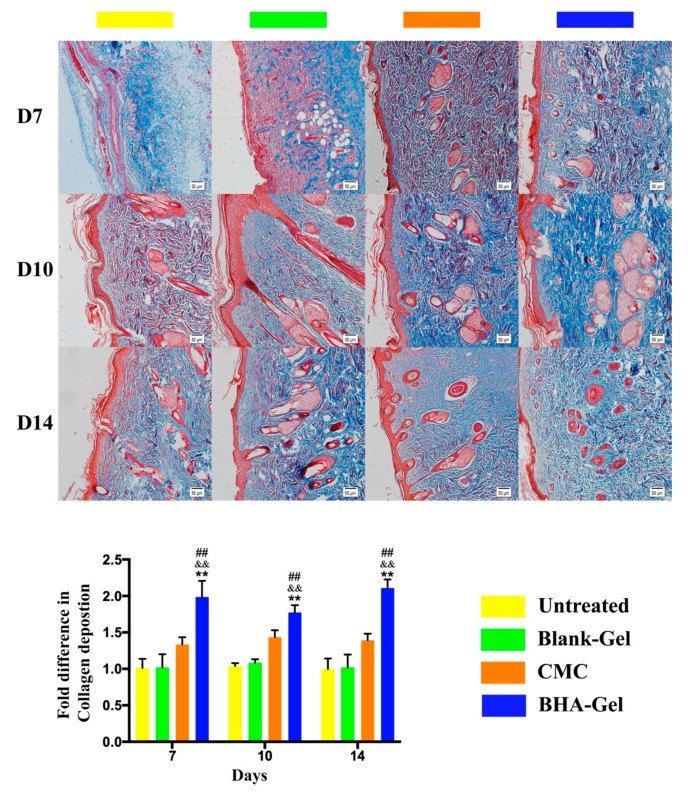
In representative Masson’s trichrome staining images (×100, bar in the lower right corner = 50 μm) (red = keratin, muscle fibers or cytoplasm, blue = collagen), the collagen deposition was quantified (*n* = 6). ## *p* < 0.01 relative to the untreated control group; && *p <* 0.01 relative to Blank-Gel; ** *p* < 0.01 relative to CMC.

The production of collagen by fibroblasts (one of the most abundant cell types in wounds [19]), as one prerequisite for the new connective tissue matrix [20], is promoted by the stimulation of potent growth factors (e.g., Transforming growth factor beta 1, TGF-β1) released from macrophages (particularly the M2 subset, see discussion below) [21]. As shown in Figure 5a, the TGF-β1 expression in wounds is significantly improved by BHA-Gel relative (*p* < 0.01). In addition, the BHA-Gel significantly increases the expression of Smad2 and Smad3 (two downstream targets of TGF-β1 [22]) (*p* < 0.05 and *p* < 0.01) (Figure 5b), whereas the expression of Smad7, a member of Smad family that deactivates Smad2 and Smad3, is significantly downregulated accordingly (*p* < 0.01) (Figure 5b). As a result, the collagen deposition, when analyzed using the Masson’s trichrome staining assay, was significantly elevated in dermal samples from the BHA-Gel group relative to other control groups (*p* < 0.01) (Figure 6).

**Figure 7:**
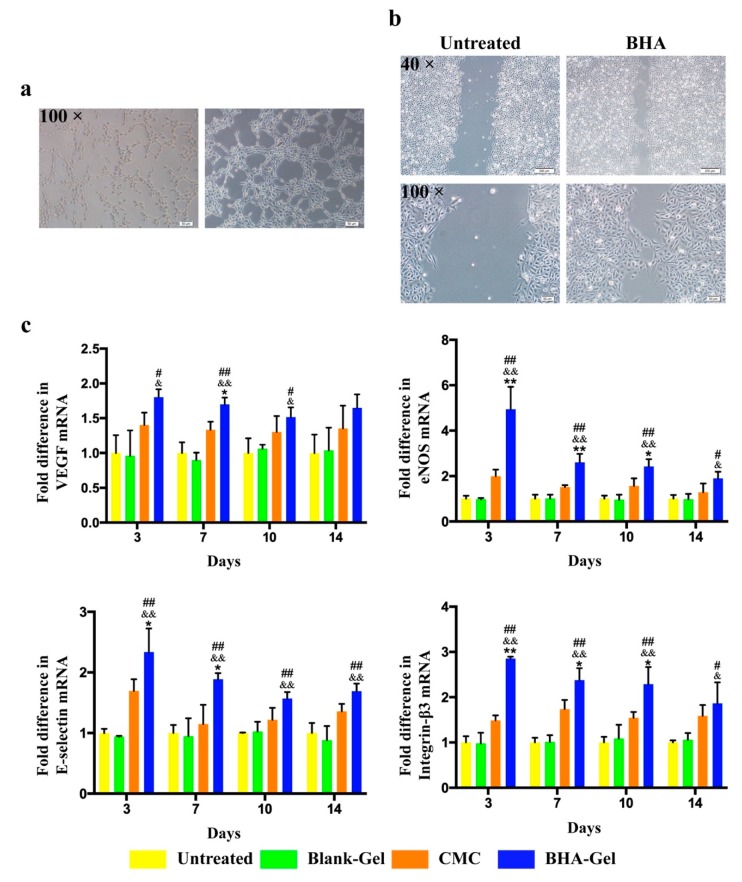
(**a**) The potential of *N*-butyrylated LMW-HA (BHA) to promote angiogenesis was assessed in Human Umbilical Vein Endothelial Cells (HUVEC), using an endothelial cell tube formation assay (×100, Bar in the lower right corner = 50 μm). (**b**) The potential of BHA to promote migration was assessed in HUVEC using the scratch assay (×40, Bar in the lower right corner = 200 μm; ×100, Bar in the lower right corner = 50 μm). (**c**) The vascular endothelial growth factor (VEGF), endothelial nitric oxide synthase (eNOS), E-selectin and Integrin-β3 mRNA levels were determined by RT-PCR (*n* = 6). # *p* < 0.05 and ## *p* < 0.01 relative to untreated control group; & *p <* 0.05 and && *p <* 0.01 relative to Blank-Gel; * *p* < 0.05 and ** *p* < 0.01 relative to CMC.

As shown in Figure 7a, BHA was able to significantly promote the tube formation of Human Umbilical Vein Endothelial Cells (HUVEC). In addition, the migration of HUVEC was also significantly promoted with the treatment of BHA (Figure 7b). As HUVEC have been commonly used for the characterization of new blood vessel formation [23,24], these in vitro results suggest that BHA is able to promote angiogenesis (Figure 7a,b).

Indeed, BHA-Gel significantly enhances the expression of the vascular endothelial growth factor (VEGF) (a growth factor for angiogenesis) in wounds (*p* < 0.05 and *p* < 0.01) (Figure 4c). In addition, a group of adhesion molecules that are required for increasing the endothelial cell proliferation and migration in wound repair, including endothelial nitric oxide synthase (eNOS) [23], E-selectin [24] and integrin-β3 [25], are also significantly upregulated by BHA-Gel (*p* < 0.05 and *p* < 0.01) (Figure 7c).

**Figure 8:**
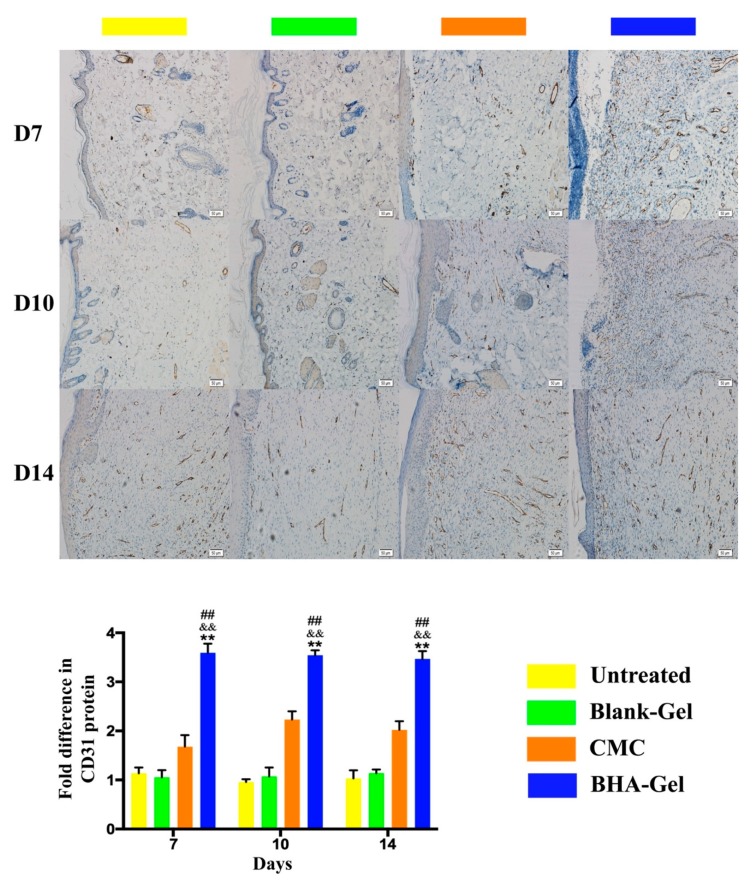
In representative immunohistochemical staining images (100×, bar in the lower right corner = 50 μm), the expression of CD31 was quantified (*n* = 6). For clarity, the expression of CD31 in the blood vessels and capillaries was not indicated by arrows in the images. ## *p* < 0.01 relative to untreated control group; && *p <* 0.01 relative to Blank-Gel; ** *p* < 0.01 relative to CMC.

**Figure 9:**
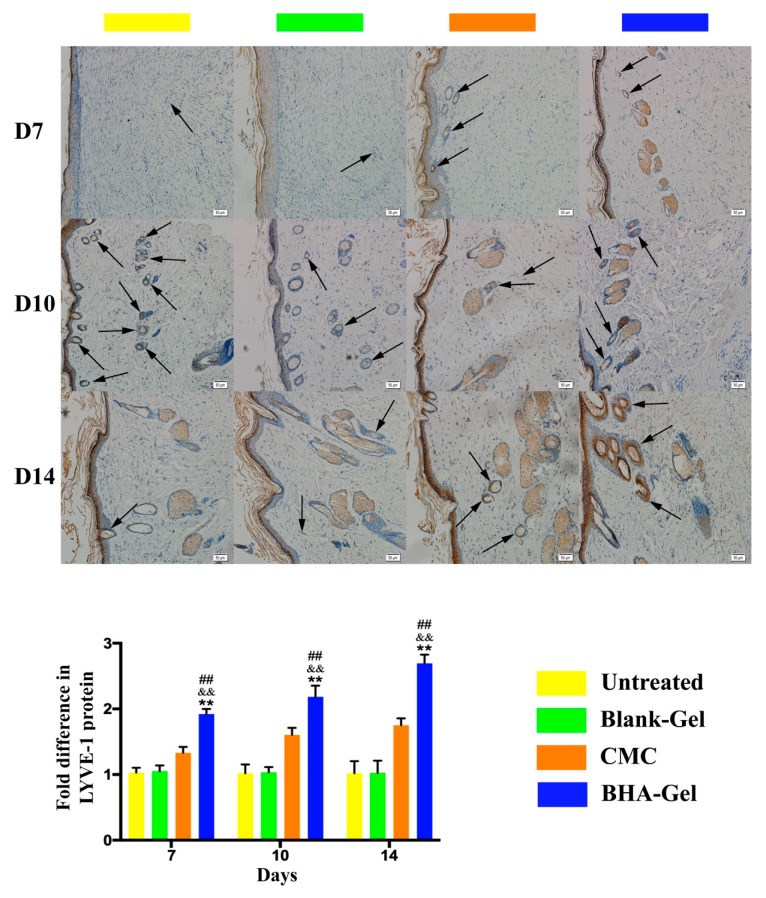
In representative immunohistochemical staining images (100×, bar in the lower right corner = 50 μm), the expression of lymph vessel endothelial hyaluronan receptor-1 (LYVE-1) on lymph vessels (indicated by arrows) was quantified (*n* = 6). ## *p* < 0.01 relative to untreated control group; && *p <* 0.01 relative to Blank-Gel; ** *p* < 0.01 relative to CMC.

In addition, the expression of CD31 (a marker for neovascularization [26]) was assessed in wounds using an immunohistochemical staining assay (Figure 8). Results indicate that BHA-Gel significantly promotes the expression of CD31 (*p* < 0.01), further confirming the role of BHA in facilitating the angiogenesis during wound healing. Moreover, the lymphatic endothelium formation (lymphangiogenesis) was examined in terms of lymph vessel endothelial hyaluronan receptor-1 (LYVE-1, a marker for lymphatic vessels [27]) expression (Figure 9). Results show that a greater level of LYVE-1 was observed in wounds treated with BHA-Gel (*p* < 0.01), indicating that BHA is able not only to promote angiogenesis but also to enhance the formation of lymphatic vessels in wound healing [28].

### 2.4. BHA Promotes Remodeling of Collagens Type III and Type I

**Figure 10:**
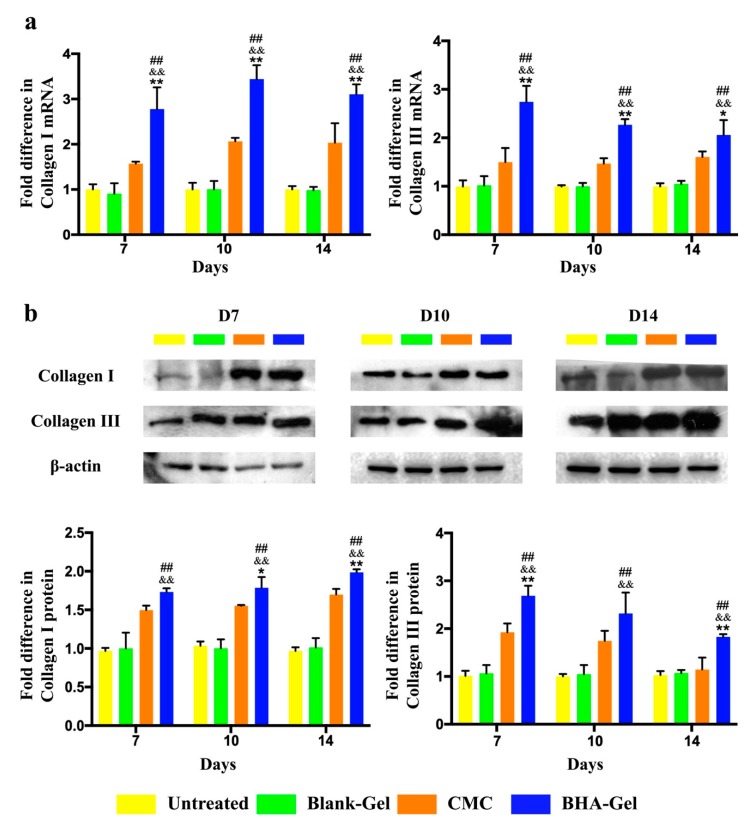
The mRNA (**a**) and protein (**b**) levels of collagen I, collagen III and β-actin were determined using RT-PCR and western blotting and quantified (*n* = 6). ## *p* < 0.01 relative to untreated control group; && *p <* 0.01 relative to Blank-Gel; * *p* < 0.05 and ** *p* < 0.01 relative to CMC.

It has been reported that TGF-β1 promotes the synthesis and accumulation of ECM proteins by activating the Smad signaling pathway [29]. Following the activation of TGF-β1/Smad-dependent pathway (Figure 5), BHA-Gel was able to significantly promote the expression of type III and type I collagens in wounds (*p* < 0.01, Figure 10).

In addition, following the treatment of BHA-Gel, the level of type III collagen expression in wounds is slowly decreased, whereas the level of type I collagen expression is gradually increased under the same conditions (Figure 10).

**Figure 11:**
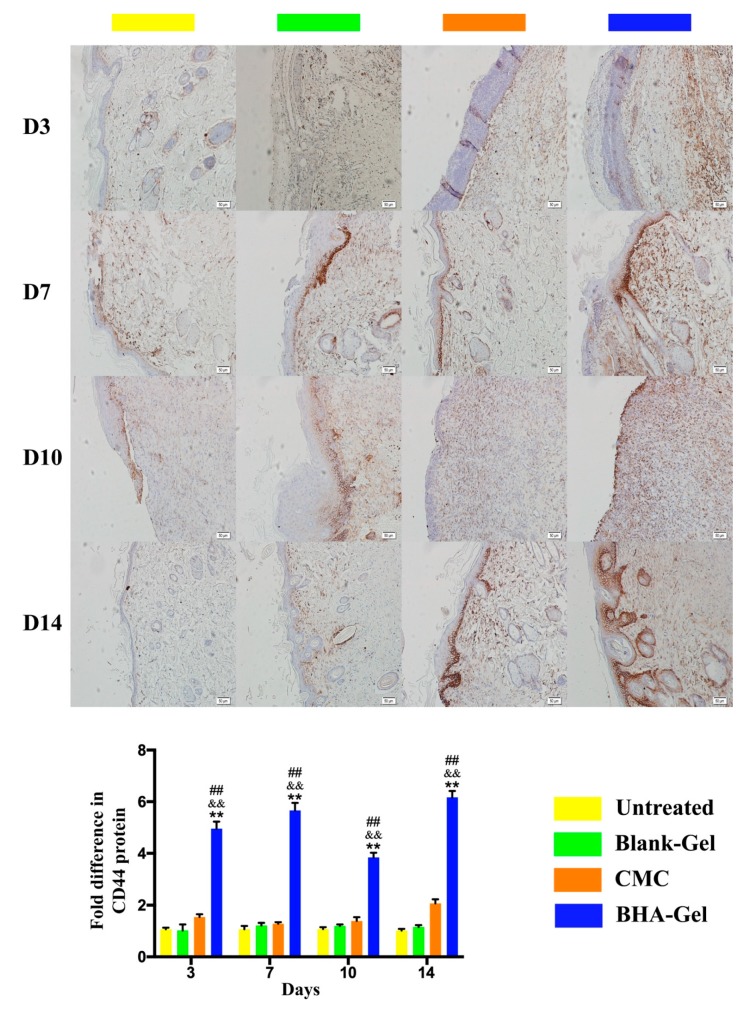
In representative immunohistochemical staining images (×100, bar in the lower right corner = 50 μm), the expression of CD44 was quantified (*n* = 6). ## *p* < 0.01 relative to the untreated control group; && *p <* 0.01 relative to Blank-Gel; ** *p* < 0.01 relative to CMC.

The expression of CD44 (a transmembrane glycoprotein widely found on diverse cell types, e.g., cutaneous fibroblasts, macrophages and endothelial cells) was significantly upregulated by BHA-Gel (*p* < 0.01) (Figure 11), implying that BHA regulates the aforementioned activities of dermal fibroblasts, macrophages and endothelial cells, mostly due to the upregulation of CD44 (see discussion below).

## 3. Discussion

As the dominant pro-inflammatory cells during the early stages of wound healing, neutrophils and M1 macrophages regulate local and systemic defense responses to the wound [30]. For example, neutrophils produce high levels of reactive oxygen species (ROS), proteases and pro-inflammatory cytokines in order to clean the wound [31]. Macrophages differentiate into the M1 subtype for phagocytic activity and the production of pro-inflammatory mediators [32].

When the inflammation phase is complete, these neutrophils go through apoptosis and become phagocytosed by the M1 macrophages, and then these M1 macrophages also phagocytose bacteria and debris to sanitize the injured site [33]. Recently, it has been reported that increased pro-inflammatory cells prolong the inflammatory response and delay the healing process, thus causing non-healing (chronic) wounds that are often evident with a deregulation of pro-inflammatory cytokines [34,35,36]. It is known that the HMW-HA produced in ECM, under normal non-inflammatory conditions, does not directly trigger signaling pathways in dendritic cells (DCs) or macrophages [37]. Following the tissue injury, the fragmentation of endogenous HMW-HA occurs as a result of the ROS release. The fragmented HA in turn enhances the inflammatory/immunostimulatory responses, as the fragments further augment the inflammation at the injured sties [38]. In contrast to conventional LMW-HA that can trigger inflammation and immunostimulation [39,40,41], BHA demonstrates anti-inflammatory activities by modulating the cytokine expression (Figure 3), indicating the capacity of BHA for preventing the wound from being trapped in a chronic inflammatory state.

It has been reported that TLR4 (a transmembrane protein belonging to the toll-like receptor family [42]) is highly activated during the early stages of wound healing and regulates pro-inflammatory cytokine production at the sites of injury [43]. As indicated above, we had demonstrated that BHA likely exerts the anti-inflammatory effects partially through the TLR4 receptor in vitro [18]. It has been reported that LMW-HA may mediate the immunostimulatory effects via the activation of NF-κB [44] (one of the transcription factors activated by TLR4). However, it is interesting to note that BHA demonstrated anti-inflammatory effects by modulating the expression of phosphorylated TAK-1 protein (Figure 4a), nuclear translocation of p65 protein (Figure 4b), and the activation of p38 MAP kinase (Figure 4c). TAK-1 is known to act as a key mediator in TLR4-mediated signaling pathways [45], and the phosphorylation of TAK-1 results in a TAK-1-dependent activation of the NF-κB and MAPK signaling pathways [46]. Therefore, these results suggest that the in vivo immunosuppressive functions of BHA result partially from the downregulation of TLR4-mediated NF-κB and MAPK signaling pathways.

The transformation from M1 to M2 macrophages plays a key role in the healing progression from the inflammatory to the proliferative phase [30]. The M2 macrophages facilitate the production of anti-inflammatory mediators, the initiation of fibroblast proliferation, and angiogenesis [33]. In this study, the production of pro-inflammatory cytokines (e.g., TNF-α, IL-1β and IL-6) associated with M1 macrophages are significantly reduced by BHA (Figure 3), while the expression of growth factors (e.g., TGF-β and VEGF) associated with M2 macrophages is significantly upregulated by BHA (Figure 5 and Figure 7c). These results suggest that M2 macrophages become dominant over the M1 subtype following the treatment of BHA, suggesting the transformation from M1 to M2 subtype at the proliferative phase [31,32,33].

When the TGF-β1 expression is significantly enhanced by BHA, the TGF-β1/SMAD-dependent pathway, which may promote wound healing via the production of ECM (a key process contributing fibrosis for the re-epithelialization [19]), is activated (Figure 5). This demonstrates the ability of BHA in the synthesis of the new collagen matrix and the resurfacing of a wound during the re-epithelialization process. Consequently, the collagen deposition is significantly elevated in wounds following the treatment of BHA-Gel (Figure 6).

Along with the re-epithelialization, there is restoration of the vascular network [36]. In this study, the production of VEGF and adhesion molecules has been significantly enhanced by BHA-Gel (Figure 7c). Moreover, the neovasculature marker CD31 is significantly upregulated with BHA-Gel (Figure 8), further confirming the role of BHA in facilitating the angiogenesis during wound healing.

In addition to angiogenesis, BHA-Gel also significantly enhances the lymphangiogenesis (Figure 9), which is evident with the upregulation of LYVE-1. Therefore, these results indicate that BHA can facilitate the angiogenesis and lymphangiogenesis during the proliferative phase of wound healing.

In the later proliferation phase, the granulation tissue is formed on the wound surface via the interplays between fibroblasts, inflammation cells and epithelial cells [32]. The granulation tissue in turn creates a framework for these cells, and regulates the proliferation, differentiation and migration of these cells within it [47,48]. These favorable behaviors were achieved by BHA, including the modulation of inflammatory responses (Figure 3 and Figure 4), the formation of extracellular collagens (Figure 5 and Figure 6) and the development of new vascular systems (Figure 7, Figure 8 and Figure 9), suggesting the formation of granulation tissues for efficient wound closure (Figure 1).

The ECM remodeling starts after the formation of granulation tissues and results in the reorganization of connective tissue [32,49]. Type III collagen, which is mainly produced in the proliferation phase, plays a key role in fibrillogenesis (the development of collagen fibrils in connective tissue) [50] and in regulating the collagen fibril diameter [51]. In addition, type I collagen as the most abundant collagen in the skin, is known to enhance the skin structure and its integrity during the maturation phase [49]. The switch from collagen type III to type I is one hallmark in ECM remodeling, which is accomplished by the interactions between fibroblasts, macrophages and endothelial cells [1]. As shown in Figure 10, the type III collagen level in wounds was slowly decreased, whereas the type I collagen level was gradually increased under the same conditions. These results imply that BHA may facilitate the dynamic remodeling of ECM (a favorable process for tissue fibrosis during the wound healing process [52]) by regulating the balance in the ratio between collagen type III and type I.

The collagen remodeling normally ends up with the formation of scar tissues (hypertrophic scar or keloid) in adult skin. It is known that a delayed wound repair is strongly associated with scarring [53], thus requiring therapeutic strategies for accelerating the wound healing and reducing the scar formation. BHA, due to the promise for accelerated wound healing, demonstrates less epidermal hyperplasia (Figure 2). In addition, scarless healing has been observed in the fetuses of mammals (e.g., mice, rats, monkeys and humans) [53], which is likely due to the absence of inflammation in fetal wounds leading to the scarless repair [54,55]. Therefore, the acceleration of skin repair taken together with anti-inflammatory functions (Figure 3) suggest that BHA may be able to attenuate the scar formation at the injured sites.

CD44, a major cell surface receptor expressed on different cell types (e.g., leukocytes, myeloid cells, fibroblasts and endothelial cells), is known to regulate cell-cell and cell-matrix interactions during wound healing [56]. For example, fibroblast migration can be mediated by CD44-dependent pathways [57], and the downregulation of CD44 does impair fibrotic activities during wound healing [56]. In addition, CD44 may facilitate the accumulation of M2 macrophages and deposition of ECM during the maturation of arteriovenous fistulae (an abnormal connection or passageway between the artery and vein) [58]. Also, it is well established that CD44 is positively involved in the neovascularization [59,60]. In this study, the expression of CD44 is significantly upregulated with BHA-Gel (Figure 11), suggesting that BHA promotes the aforementioned activities of dermal fibroblasts, macrophages and endothelial cells, partially due to the upregulation of CD44.

It is worth noting that DHA and AHA significantly delays the dermal wound repair (Figures S4 and S5). It is likely due to the fact that DHA and AHA trigger the production of pro-inflammatory cytokines, delaying the inflammation phase of the wound healing (Figures S6 and S7), although they demonstrate the potential of re-epithelialization and angiogenesis (Figures S8 and S9). In contrast, when the naturally-occurring *N*-acetyl group of HA is replaced with the longer *N*-acyl chain of the *N*-butyryl group, BHA, the partially *N*-butyrylated LMW-HA significantly accelerates skin repair at a lower dose (0.25 mg/mL), when compared to a commercial wound care product (containing 5 mg/mL carboxymethyl chitosan). These results therefore suggest the critical role of *N*-acylation of LMW-HA in wound healing.

Recently, a combination of HA with different molecular weights and silver nanoparticles has improved the wound healing in older rats and rats with diabetes [61]. Due to the beneficial effects on different stages during the wound healing process, BHA also has therapeutic potential for chronic wounds. Therefore, the application of BHA alone or in combination with other therapeutic modalities, to promote healing efficiency in animal models with chronic wounds, will be performed in future.

## 4. Materials and Methods

### 4.1. Wound Healing Efficacy

The animal ethics committee of Jilin University approved all of the experiments (the approval number: 20180010; 1 January 2018). All animals received care in compliance with the guidelines outlined in the Guide for the Care and Use of Laboratory Animals. Male Wistar rats (~200 g, purchased from the Changchun Institute of Biological Products, China) were maintained in a pathogen-free animal facility for two weeks prior to the experiments (Figure S10). The wound healing efficacy was assessed using rats with full-thickness wounds (see the Supporting Information).

### 4.2. Therapeutic Mechanisms

The potential of angiogenesis and migration were examined in HUVEC (Human Umbilical Vein Endothelial Cells) using the Matrigel-based (a liquid laminin/collagen gel) Endothelial Cell Tube Formation Assay [62] and the Scratch Assay [63] (see Supporting Information). In vivo, the determination of mRNA and protein expression was performed using RT-PCR, western blotting and ELISA (see Supporting Information). In addition, histopathological examinations were performed using the hematoxylin-eosin (H&E), Masson’s trichrome and immunohistochemical staining assays (see the Supporting Information).

### 4.3. Statistical Analysis

Data were calculated as the mean ± standard deviation (SD). The log rank test was used for comparison in survival studies. An unpaired Student’s *t*-test (two-tailed) was used to test the significance of the differences between two mean values. A two-way Analysis of Variance (ANOVA) (Bonferroni’s Post-Hoc test) was used to test the significance of the differences in three or more groups. In all experiments, *p* < 0.05 was considered statistically significant.

## Supplementary Materials

Supplementary materials can be found at https://www.mdpi.com/1422-0067/20/15/3722/s1.

## Abbreviations

HMW-HA	High molecular weight hyaluronic acid
LMW-HA	Low molecular weight hyaluronic acid
ECM	Extracellular matrix
CMC	Carboxymethyl chitosan
TNF-α	Tumor necrosis factor-α
IL-6	Interleukin 6
MAP3K7	Mitogen-activated protein kinase, kinase 7
TGF-β1	Transforming growth factor beta 1
TAK-1	TGF-β activated kinase 1
NF-κB	Nuclear factor-κB
MAPK	Mitogen-activated protein kinases
LYVE-1	Lymph vessel endothelial hyaluronan receptor-1
ROS	Reactive oxygen species
