# Supplementary material for: A Low Molecular Weight Hyaluronic Acid Derivative Accelerates Excisional Wound Healing by Modulating Pro-Inflammation, Promoting Epithelialization and Neovascularization, and Remodeling Collagen
Source: Int J Mol Sci. 2019 Jul 30;20(15):3722. doi: 10.3390/ijms20153722 (PMC6695899; doi:10.3390/ijms20153722)
Supplement: Supplementary file 1 [file ijms-20-03722-s001.pdf]

International Journal of *Molecular Sciences*

MDPI

*Supporting Information*

# A Low Molecular Weight Hyaluronic Acid Derivative Accelerates Excisional Wound Healing by Modulating Pro-Inflammation, Promoting Epithelialization and Neovascularization, and Remodeling Collagen

## 1. Materials and Methods

### *1.1. Preparation and Characterization of LMW-HA Derivatives*

The low molecular weight hyaluronic acid (LMW-HA) derivatives were synthesized and characterized as described previously [1]. The LMW-HA solutions were prepared in sterilized ultrapure water, and the endotoxin level in solutions was measured using an Endotoxin Assay kit (GenScript, Piscataway, NJ, USA) according to the manufacturer's instructions.

In addition, a gel formulation was prepared for in vivo studies. Briefly, 30 g of alginate, 450 g of glycerin, 2 g of ethylparaben, and 0.5 g of calcium gluconate were prepared in 1 L of sterilized ultrapure water to produce the Blank-Gel. In addition, LMW-HA solutions prepared as described above were added into the Blank-Gel to form the LMW-HA-Gel.

### *1.2. Wound Healing Efficacy*

Four excisional full-thickness wounds (~1.8 $cm^2$) per animal were made with disinfected surgical scissors deep into the dermis, without damaging the subdermal vasculature on the dorsal surface (Day 0) (Figure S10). On the same day, the wounds were treated daily with or without 0.2 mL of LMW-HA-Gel ([c] = 0.05, 0.1, 0.25, 0.5 and 1 mg/mL), and the wound diameter was measured at Day 3, 7, 10 and 14 (Figure S3). The wound closure rate (6 wounds per group) was calculated as $(1\text{-}S_n/S_0) \times 100\%$, where Sn = the wound surface area at a predetermined day, thus, $S_0$ = the wound surface area at Day 0.

In addition, four excisional full-thickness wounds (~1.8 $cm^2$) per animal were made as described above. From Day 0, the wounds were treated daily with 0.2 mL of Blank-Gel (negative control group), carboxymethyl chitosan (CMC) ([c] of CMC = 5 mg/mL in CHITIN®, a commercial wound care product purchased from Shijiazhuang Yishengtang Medical Supplies Ltd., Shijiazhuang, China) (positive control group), and LMW-HA-Gel ([c] of LMW-HA = 0.25 mg/mL) (Figure 1b), and the wound diameter was measured at Days 3, 7, 10 and 14. The wound closure rate (6 wounds per group) was calculated as described above.

### *1.3. Therapeutic Mechnisams*

#### 1.3.1. In Vitro Studies

The Human Umbilical Vein Endothelial Cells (HUVEC) cell line was purchased from the American Type Culture Collection (ATCC, USA). Cells were maintained in RPMI-1640 medium (Corning, NY, USA) containing 10% fetal bovine serum (FBS; Gibco, Waltham, MA, USA) and a Penicillin-Streptomycin Nystatin solution (Biological Industries, Israel) at 37 °C under a 5% $CO_2$ atmosphere.

Cell proliferation was examined using a Matrigel-based (a liquid laminin/collagen gel) Endothelial Cell Tube Formation Assay [2]. Briefly, 200 μL of Matrigel (Corning) per well were added into 24-well plates. When the Matrigel solidified, HUVEC were seeded at a density of $3\times 10^5$ cells per well for 24 h. Cells were then treated with 0.05 mg/mL de-acetylated LMW-HA (DHA), AHA and *N*-

butyrylated LMW-HA (BHA) in fresh growth medium for 48 h. Subsequently, these cells were observed using a microscope (Olympus BX53, Tokyo, Japan).

Cell migration was studied using the scratch assay [3]. Briefly, HUVEC were seeded in 6-well plates at a density of $4 \times 10^5$ cells per well to reach 100% confluence. The scratches were made by pipette tips through the monolayer in the middle of the plate. Cells were then treated with serum-free medium containing LMW-HA derivatives at 0.05 mg/mL. After 48 h, images were obtained using a microscope (Olympus BX53).

1.3.2. In Vivo Studies

Four excisional full-thickness wounds (~1.8 $cm^2$) per animal were made as described above, in which one wound was used as the untreated control, and three others were daily treated with 0.2 mL of Blank-Gel, CMC ([c] of CMC = 5 mg/mL), and LMW-HA-Gel ([c] of LMW-HA = 0.25 mg/mL) from Day 0. The whole wound specimen, without the surrounding healthy tissues, was collected for the following experiments (6 wounds were used on each time point per group in one experiment).

Determination of mRNA expression via reverse transcription polymerase chain reaction (RT-PCR) was performed as follows: The wounds within TriZol Up reagent (TransGen Biotech, Beijing, China) were homogenated using a tissue grinder (Scientz, Ningbo, Zhejiang, China). The homogenates were centrifuged at 10,000 g for 15 min at 4 °C to remove the insoluble debris, and the supernatant was collected for a reverse transcription polymerase chain reaction (RT-PCR). First-strand cDNA was obtained from total RNA samples using the TransScript® All-in-One First-Strand cDNA Synthesis SuperMix kit (TransGen Biotech, Beijing, China). Quantitative real-time RT-PCR was carried out using the StepOnePlus™ Real-Time PCR System (Thermo Scientific). RT-PCR was performed under the following conditions: An initial denaturation step at 94 °C for 30 s, followed by 45 cycles of 5 s at 94 °C, annealing for 30 s at 60 °C. The primers used were listed in Table S1. The quantitative level of each target mRNA was measured as a fluorescent signal, corrected according to the signal for β-actin RNA.

Determination of protein expression via western blotting and enzyme-linked immunosorbent assay (ELISA) was performed as follows: The wounds within the RIPA Lysis Buffer (GenStar, China) containing 1 mM PMSF (GenStar, China) were homogenated using a tissue grinder (Scientz, Ningbo, Zhejiang, China). The homogenates were centrifuged at 10,000 g for 15 min at 4 °C to remove the insoluble debris in order to collect the total protein. In addition, nuclear protein from the wounds was obtained using the Nuclear and Cytoplasmic Protein Extraction Kit (Beyotime, China) containing 1 mM PMSF (Beyotime, China) in accordance with the manufacturer's instructions. Protein concentrations were determined using the BCA kit (TransGen Biotech, China). 20 μg of protein per sample were loaded onto an SDS-polyacrylamide gel and electrophoresed at 100 V for 2 h. Protein was then transferred to a polyvinylidene difluoride (PVDF) membrane (Millipore) for 1.5 h at 200 mA. Membranes were incubated overnight with the appropriate primary antibodies [anti-transforming growth factor beta 1 (anti-TGF-β1)] antibody (ab179695), anti-TGF-β activated kinase 1 (anti-TAK-1) antibody (ab109526), anti-p-TAK-1 antibody (ab109404) and anti-p38 antibody (ab107799), Abcam, USA; Anti-p-p38 antibody (AF4001), anti-Collagen I antibody (AF7001), anti-Collagen III antibody (AF0136), anti-β-actin antibody (AF7018), Affinity, USA] at 4 °C. Antibody reactive bands were detected with HRP-labeled secondary antibodies using the EasySee® Western Blot kit (TransGen Biotech, China). In addition, the concentrations of tumor necrosis factor-$\alpha$ (TNF-$\alpha$), IL-6 and interleukin-1β (IL-1β) were determined using the Rat TNF-$\alpha$ ELISA kit, Rat interleukin 6 ELISA kit, and Rat interleukin 1β ELISA kit (Cusbio, China).

Histopathological examinations were performed as follows: The wound biopsies were fixed in 4% paraformaldehyde, embedded in paraffin, and sectioned (4 μm). Sections were treated with hematoxylin-eosin (H&E) and Masson's trichrome stains, respectively.

In the H&E staining assay, the inflammatory cell infiltration, fibroblast proliferation and blood vessel formation were observed under a microscope (Olympus BX53). To quantify the inflammatory cell infiltration, the integrated optical density (IOD) and the number (n) of positive cells in slides were analyzed using Image-Pro Plus 6.0 software (Media Cybernetics, Inc., USA). The quantitative level in

the inflammatory cell infiltration with the treatment of Blank-Gel, CMC and BHA-Gel was determined as the IOD/n corrected according to the untreated control group. To quantify the fibroblast proliferation, three areas of the epidermis layer in one slide were randomly selected to measure the mean of the epidermal thickness using Image-Pro Plus 6.0 software. The quantitative level in fibroblast proliferation with the treatment of Blank-Gel, CMC and BHA-Gel was determined as the mean of the epidermal thickness corrected according to untreated control group. In addition, the development of blood vessels was quantified based on the mean of new blood vessels in slides.

In Masson's trichrome staining assay, collagen depositions were observed under a microscope (Olympus BX53). As described above, the quantitative level in the collagen deposition with the treatment of Blank-Gel, CMC and BHA-Gel was measured as the IOD/n, corrected according to the untreated control group.

In addition, the dewaxed sections were immediately immersed in 3% $H_2O_2$ to block endogenous peroxidase, and the antigen retrieval was performed using 0.01 M citrate buffer, pH 6.0 (slightly acidic) (Maxim, Fuzhou, China). The sections were then blocked in 5% BSA (Roche). Primary antibodies including anti-CD31 antibody (ab182981, Abcam, USA), anti-lymph vessel endothelial hyaluronan receptor-1 (anti-LYVE-1) antibody (NB600-1008SS, Novus) and anti-CD44 antibody (ab189524, Abcam, USA) were incubated overnight at 4°C, followed by the incubation with HRP-labeled secondary antibodies (Affinity, USA). After counterstaining with hematoxylin, positively-stained cells were observed under a microscope (Olympus BX53). As described above, the quantitative level in antigen expression with the treatment of Blank-Gel, CMC and BHA-Gel was measured as the IOD/n, corrected according to untreated control group.

**Table S1.** Primers used for quantitative reverse transcription polymerase chain reaction (qRT-PCR).

| Template | Forward Primer (5′–3′) | Reverse Primer (5′–3′) |
|---|---|---|
| TGF-β1 | ATGACATGAACCGACCCTTC | ACTTCCAACCCAGGTCCTTC |
| Smad 2 | CTGGCTCAGTCTGTCAACCA | CTGCCTCCGATATTCTGCTC |
| Smad 3 | CCAGTGCTACCTCCAGTGTT | CTGGTGGTCGCTAGTTTCTC |
| Smad 7 | GGAGTCCTTTCCTCTCTC | GGCTCAATGAGCATGCTCAC |
| Collagen I | GAGCGGAGAGTACTGGATCG | TACTCGAACGGGAATCCATC |
| Collagen III | ACCTCCTGGTGCTATTGGTC | TCTCTCCATTGCGTCCATC |
| TNF-α | GACCCTCACACTCAGATCATCTTCT | TGCTACGACGTGGGCTACG |
| β-actin | CCTTCCTGGGTATGGAATCCT | GGAGCAATGATCTTGATCTT |
| VEGF | AGGCCAGCACATAGGAGAGA | TTTCTTGCGCTTTCGTTTTT |
| eNOS | CGAGATATCTTCAGTCCCAAGC | GTGGATTTGCTGCTCTCTAGG |
| E-selectin | GTCTGCGATGCTGCCTACTTG | CTGCCACAGAAAGTGCCACTAG |
| Integrin-β3 | CTTCTCCTGTGTCCGCTACA | GAGTAGCAAGGCCAATGAGC |
| IL-6 | TAGTCCTTCCTACCCCAACTTCC | TTGGTCCTTAGCCACTCCTTC |
| IL-1β | GAAATGCCACCTTTTGACAGTG | GAAATGCCACCTTTTGACAGTG |

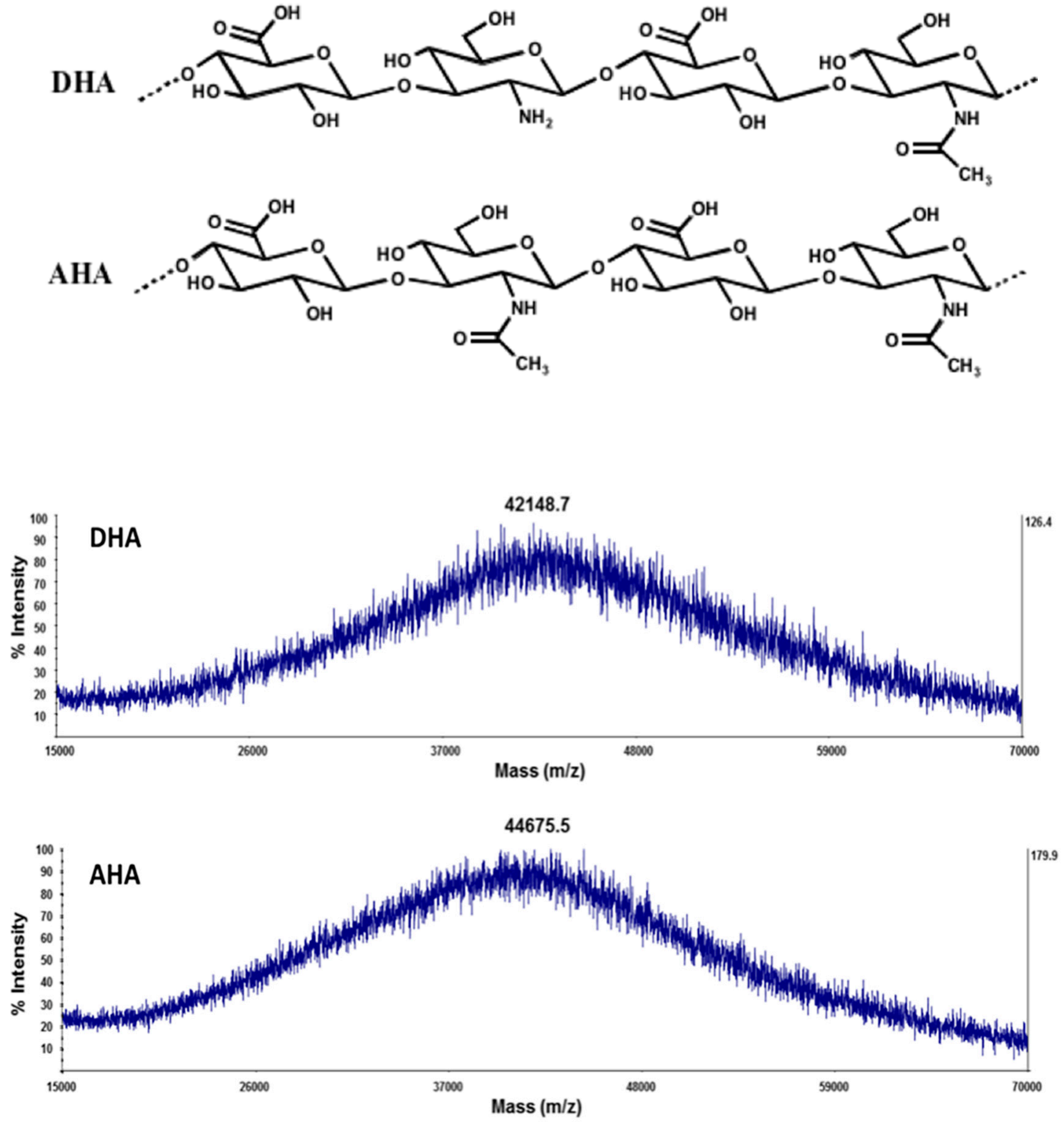


**Figure S1.** The Schematic of DHA (partially deacetylated hyaluronic acid (HA)) and AHA (reacetylated DHA). MALDI-TOF spectrum of DHA and AHA generated using DHB at the cationic matrix.

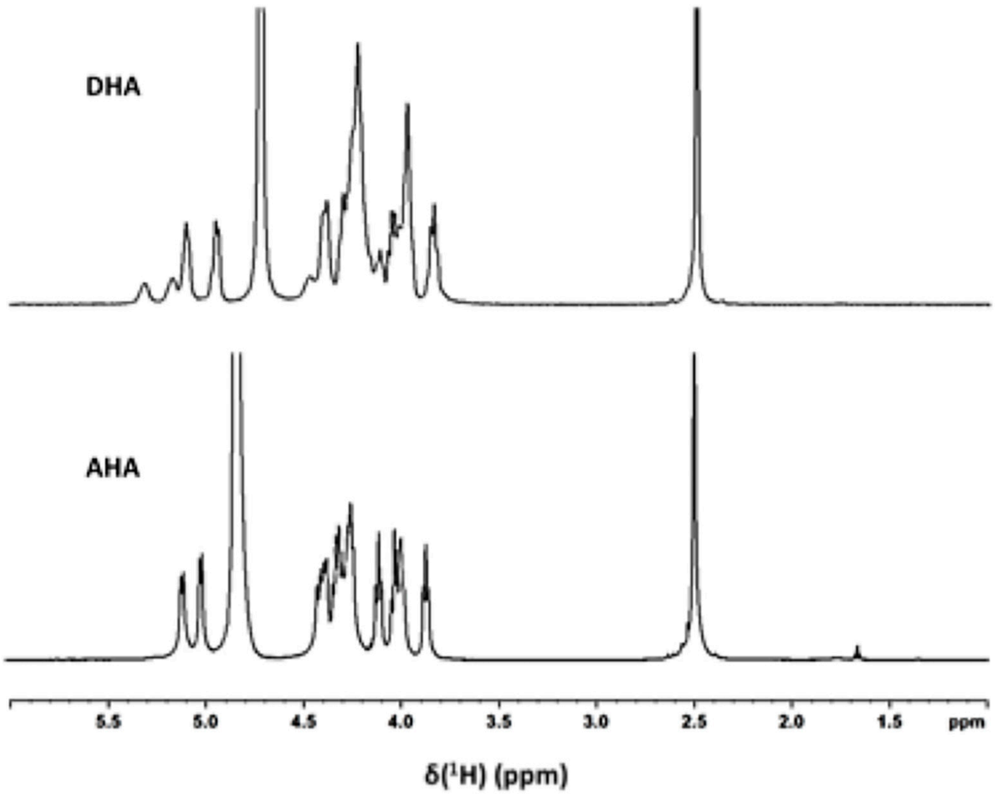


**Figure S2.** $^1$H NMR spectra of HA, DHA and *N*-butyrylated LMW-HA (BHA). Polysaccharides were prepared at a concentration of 10 mg/mL in $D_2O$. $^1$H NMR spectra of HA, DHA, AHA and BHA were recorded at 348 K with a 500 MHz spectrometer.

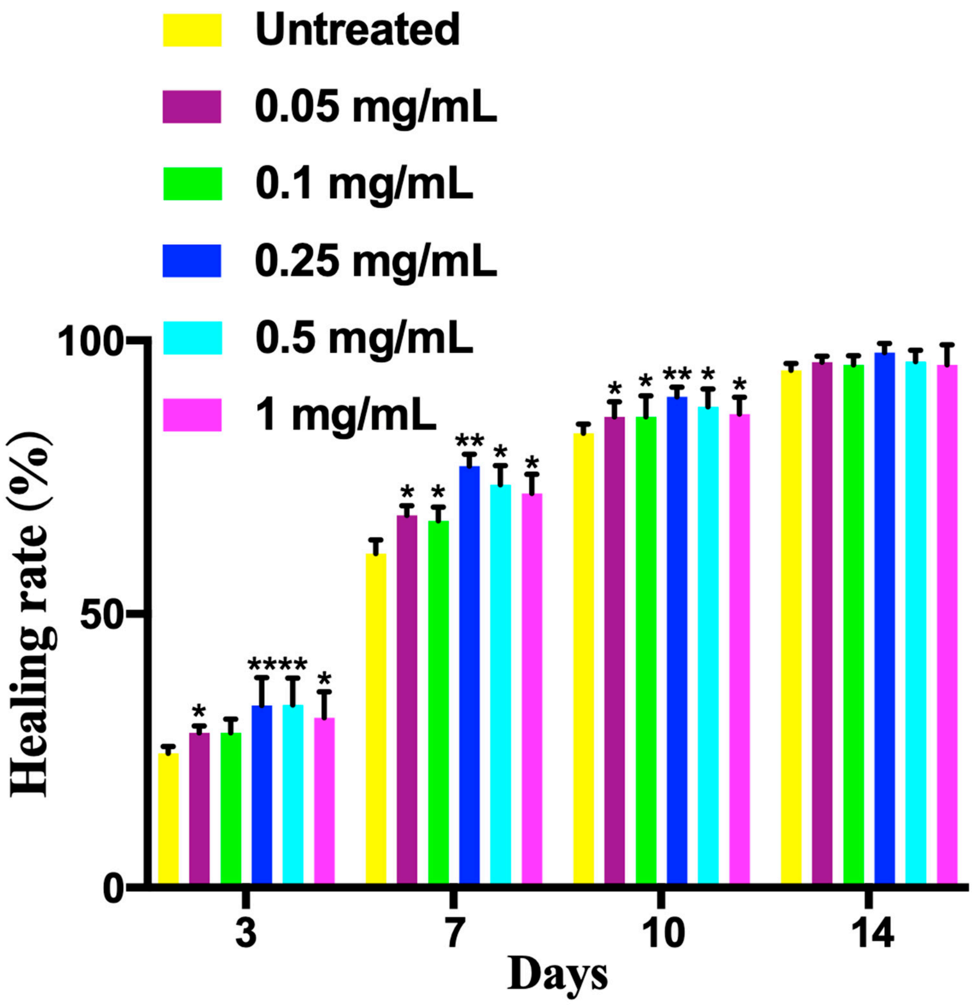


**Figure S3.** Healing rate (%) treated BHA-Gel at different concentrations on Days 3, 7, 10 and 14 when compared with the wound area on Day 0 ($n = 6$). * $p < 0.05$ and ** $p < 0.01$ relative to untreated control group.

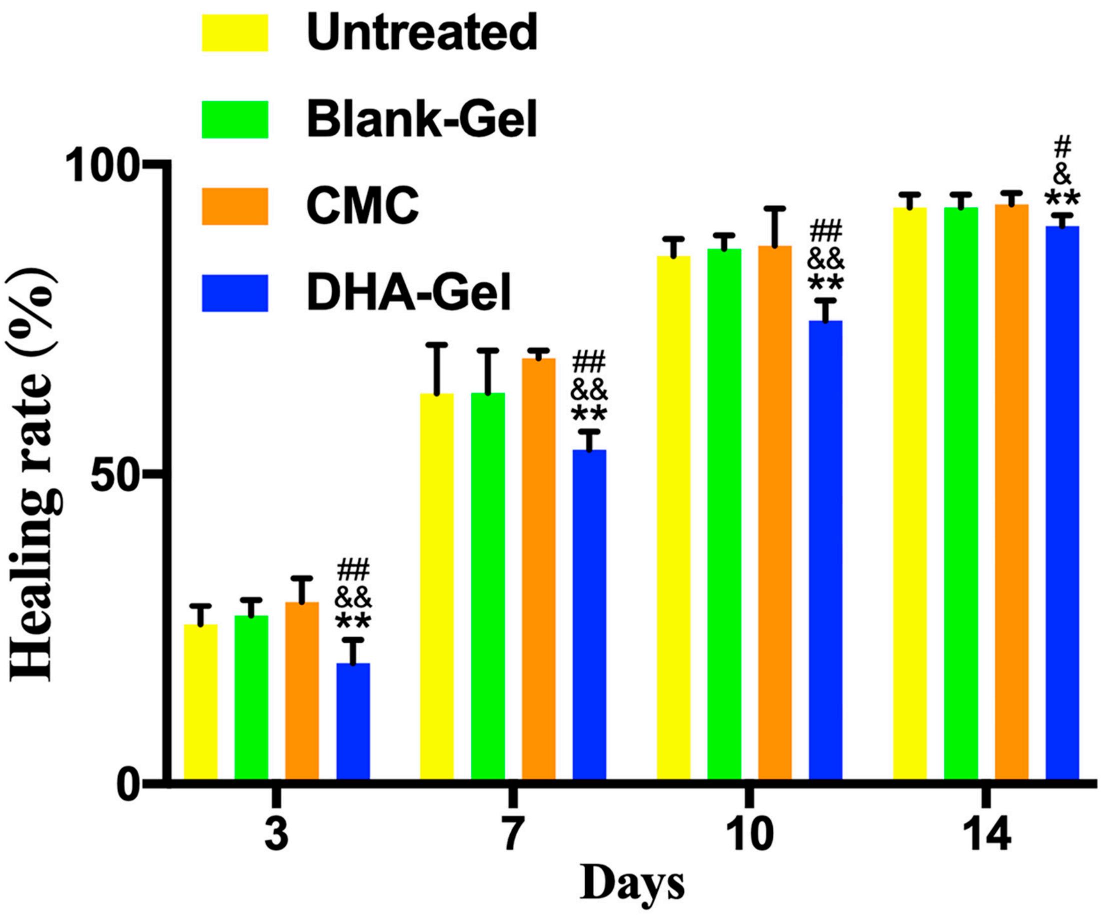


**Figure S4.** Healing rate (%) treated DHA-Gel (DHA = 0.25 mg/mL) on Days 3, 5, 7, 10 and 14 when compared with the wound area on Day 0 ($n = 6$). ## $p < 0.01$ relative to untreated control group; && $p < 0.01$ relative to Blank-Gel; * $p < 0.05$ and ** $p < 0.01$ relative to carboxymethyl chitosan (CMC).

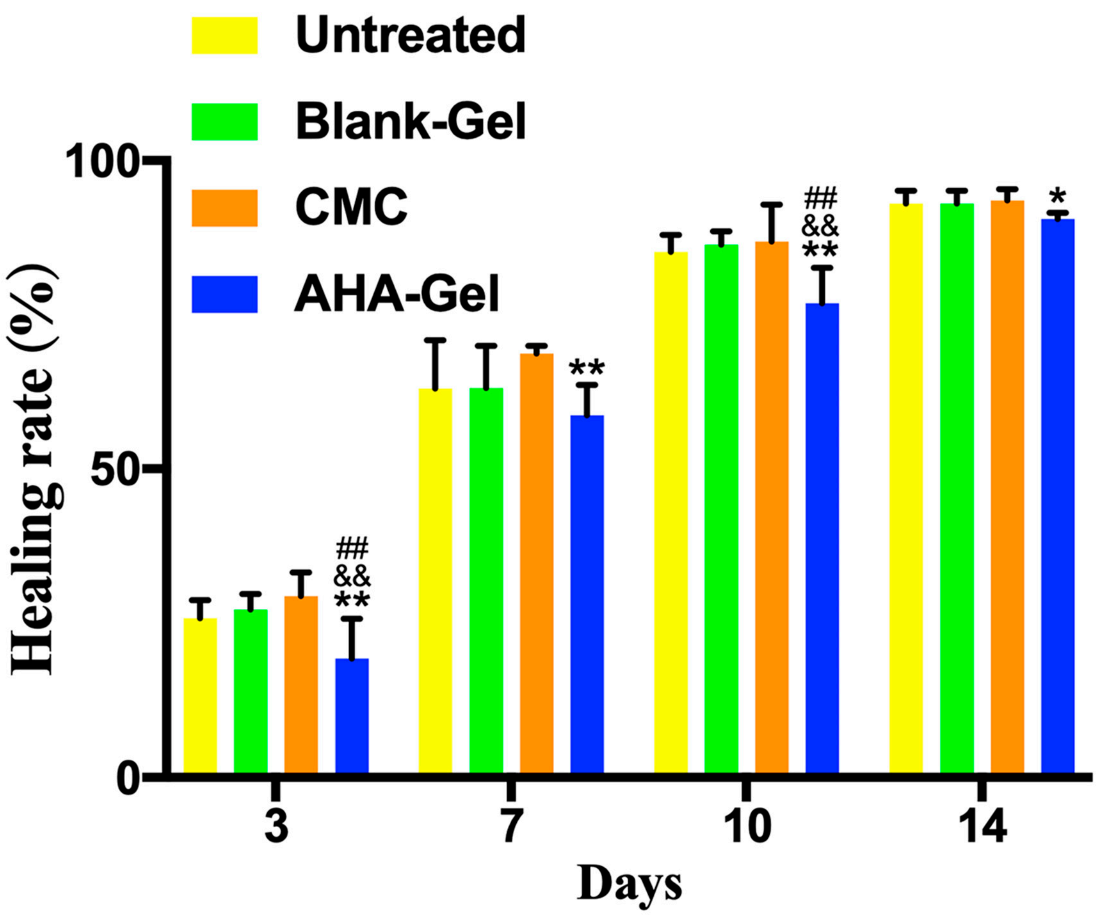


**Figure S5.** Healing rate (%) treated AHA-Gel (AHA = 0.25 mg/mL) on Days 3, 5, 7, 10 and 14 when compared with the wound area on Day 0 ($n = 6$). ## $p < 0.01$ relative to untreated control group; && $p < 0.01$ relative to Blank-Gel; * $p < 0.05$ and ** $p < 0.01$ relative to CMC.

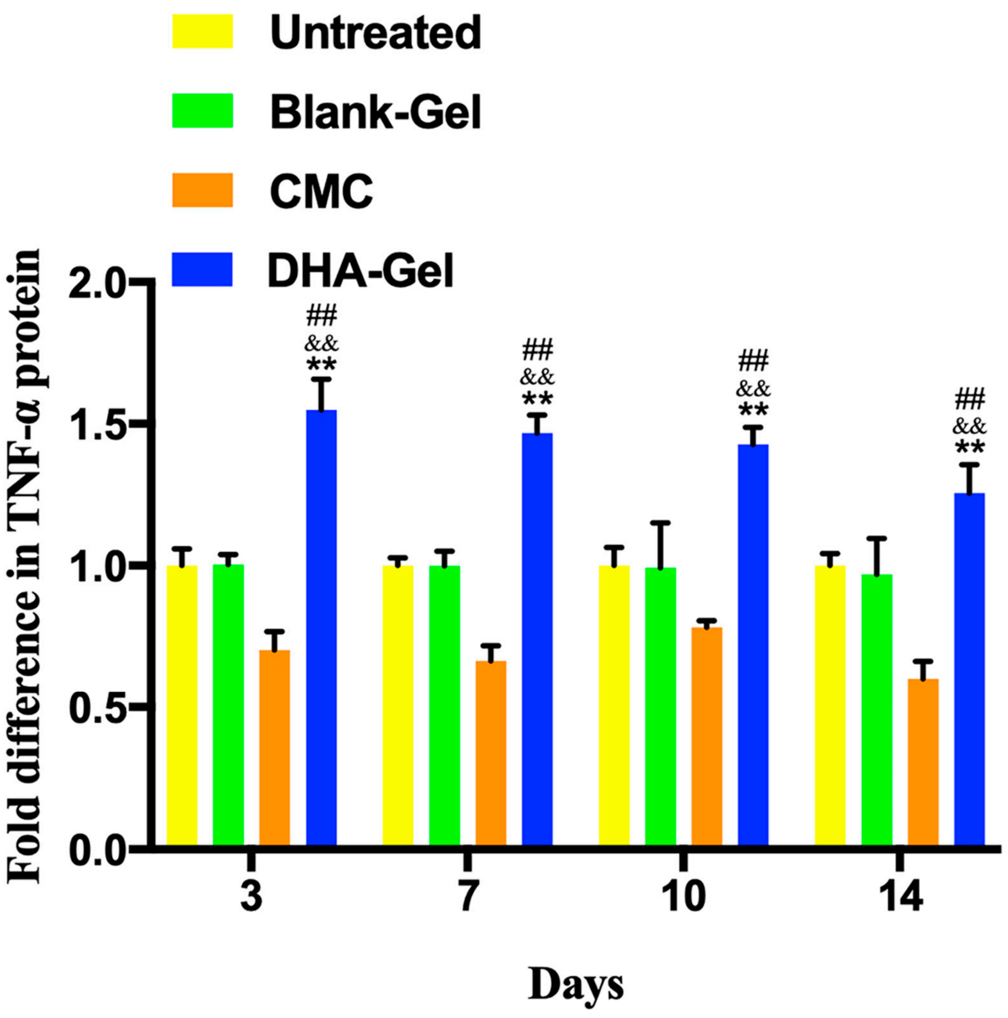


**Figure S6.** The protein level of TNF-α in wounds from rats ($n$ = 6) treated with DHA-Gel was determined by ELISA, and was shown as the fold change to those of rats in the untreated control group. ## $p < 0.01$ relative to untreated control group; && $p < 0.01$ relative to Blank-Gel; ** $p < 0.01$ relative to CMC.

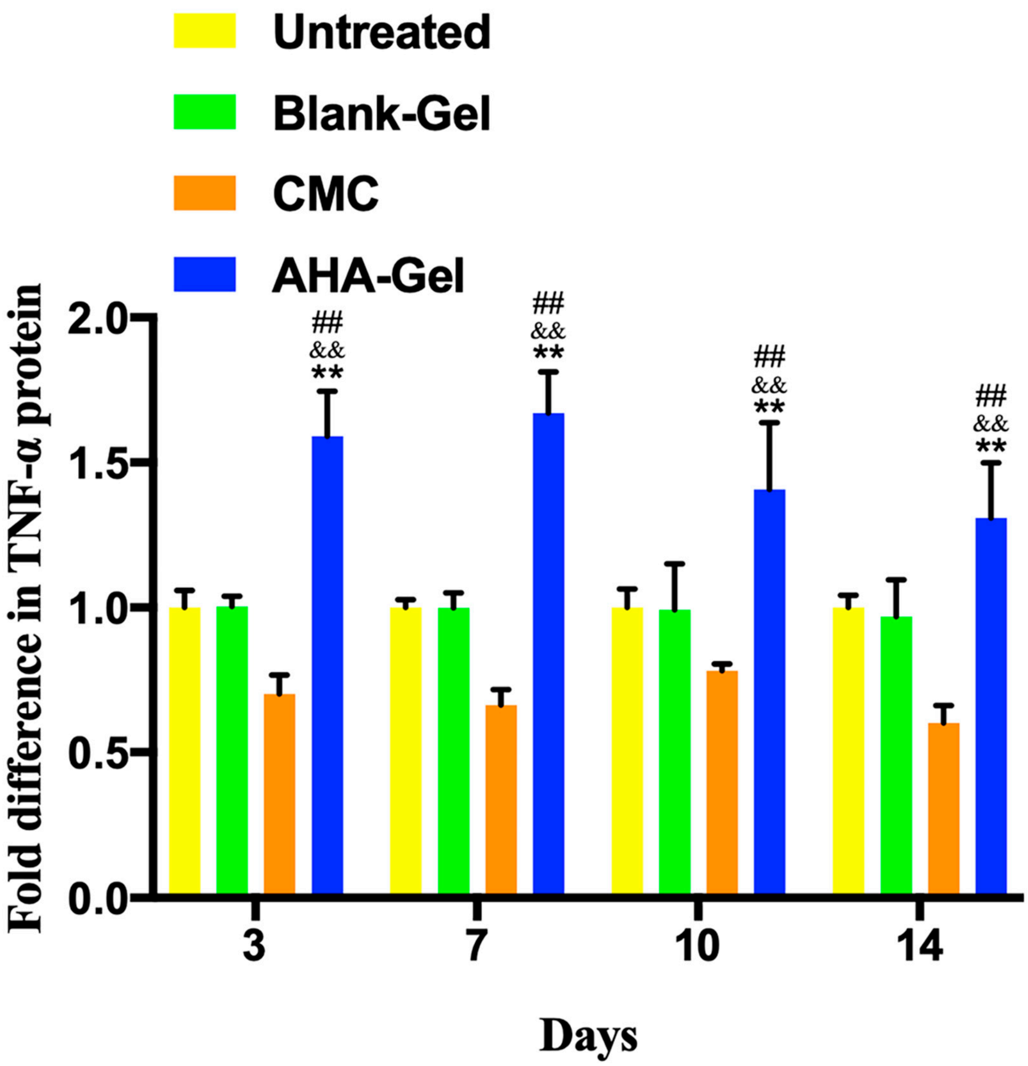


**Figure S7.** The protein level of TNF-α in wounds from rats ($n$ = 6) treated with AHA-Gel was determined by ELISA and was shown as the fold change to those of rats in the untreated control group. ## $p < 0.01$ relative to untreated control group; && $p < 0.01$ relative to Blank-Gel; ** $p < 0.01$ relative to CMC.

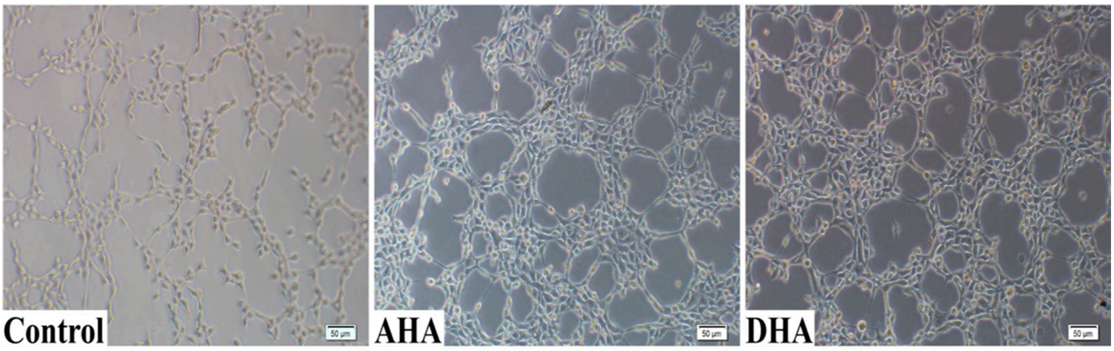


**Figure S8.** The proliferation of Human Umbilical Vein Endothelial Cells (HUVEC) was assessed with AHA and DHA using the Endothelial Cell Tube Formation Assay (100x, Bar = 50 μm).

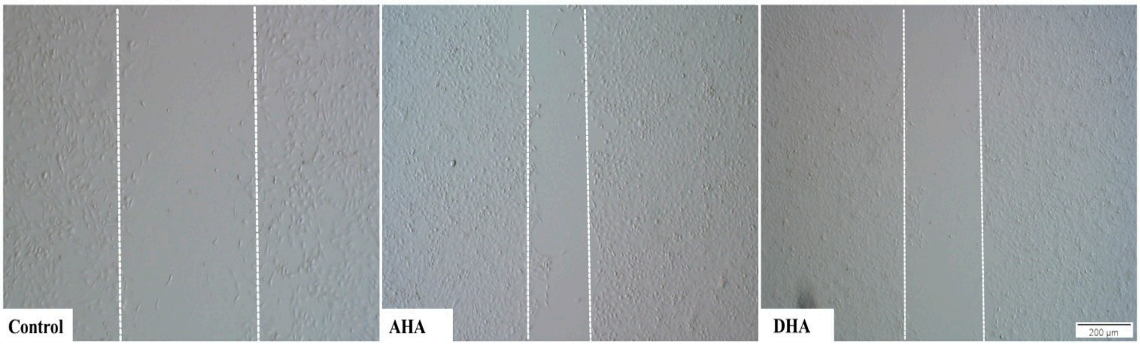


**Figure S9.** The migration of HUVEC was assessed with AHA and DHA using the scratch assay (40x, Bar = 200 μm).

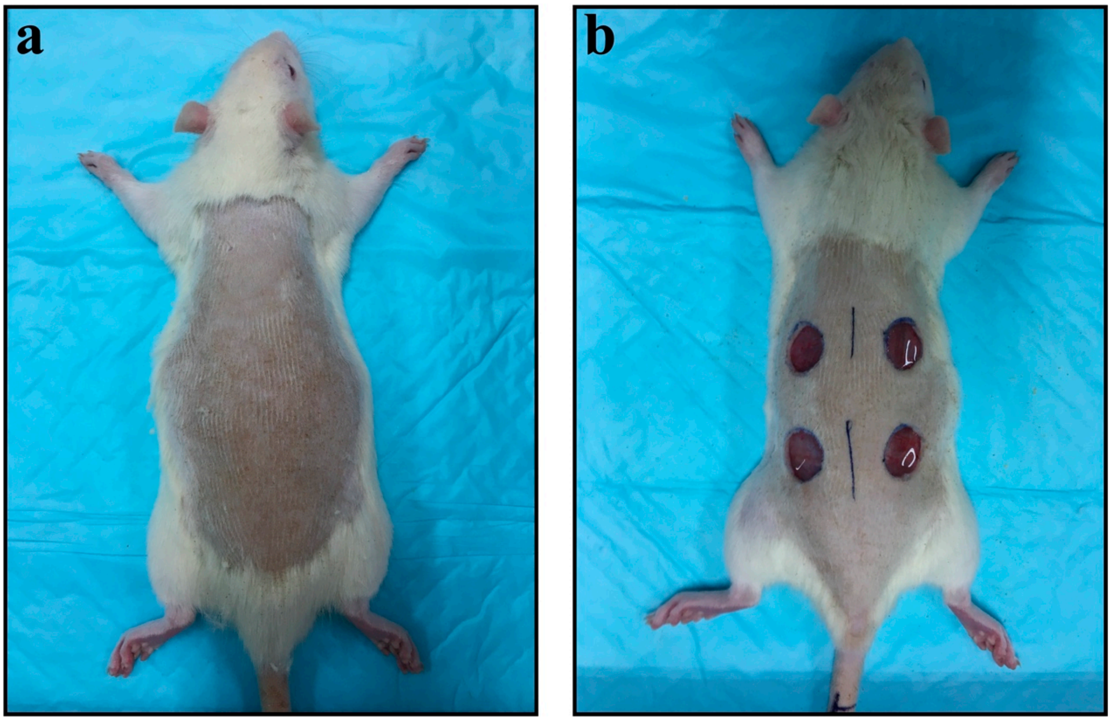


**Figure S10.** Rats (**a**) before and (**b**) after four excisional full-thickness wounds per animal.

## References

1. Babasola, O.; Rees-Milton, K.J.; Bebe, S.; Wang, J.X.; Anastassiades, T.P. Chemically modified N-acylated hyaluronan fragments modulate proinflammatory cytokine production by stimulated human macrophages. *J. Biol. Chem.* **2014**, *289*, 24779–24791.
2. Skovseth, D.K.; Kuchler, A.M.; Haraldsen, G. The HUVEC/Matrigel assay: an in vivo assay of human angiogenesis suitable for drug validation. *Methods Mol. Biol.* **2007**, *360*, 253-268.
3. Liang, C.C.; Park, A.Y.; Guan, J.L. In vitro scratch assay: a convenient and inexpensive method for analysis of cell migration in vitro. *Nat. Protoc.* **2007**, *2*, 329-333.
